# Theranostic Nanoparticles for Fluorosensitive Visualization and Inhibition of Amyloid Beta‐Induced Neuroinflammation

**DOI:** 10.1002/ppsc.202500220

**Published:** 2026-02-19

**Authors:** Umme Tamima, Hoda M. Gebril, Md Ragib Hasan, Miguel A. Quintero, Aravind Aryasomayajula, Diya Chengappa, Imran Attarwala, Bradley L. Truong, Prabhas V. Moghe, Kathryn E. Uhrich

**Affiliations:** ^1^ Department of Chemistry University of California Riverside California USA; ^2^ Department of Biomedical Engineering Rutgers University Piscataway New Jersey USA; ^3^ Cooper Medical School Rowan University Camden New Jersey USA; ^4^ Department of Chemical & Biochemical Engineering Rutgers University Piscataway New Jersey USA; ^5^ University of Texas at Dallas Richardson Texas USA

**Keywords:** amphiphilic macromolecules, fibril amyloid beta, microglia, nanoparticles, neuroinflammation, rhodamine‐tagged macromolecules

## Abstract

The emerging field of microglial therapies has significant potential to alleviate fibrillar amyloid beta (fAβ)‐associated neuroinflammation, which exacerbates neurodegeneration in Alzheimer's disease (AD). New therapeutic strategies integrate with diagnostic capabilities to robustly elucidate the mechanisms and consequences of intervention. Amphiphilic macromolecules (AMs), comprising a hydrophilic sugar backbone, hydrophobic aliphatic side chains, and poly(ethylene glycol) (PEG) segments for enhanced stability, exhibit significant potential for biomedical applications due to their biocompatibility and self‐assembled nanoscale structures. This study presents rhodamine B‐tagged (Rh) AMs (Rh‐AMs) that create stable nanoparticles (Rh‐AM‐NPs) with potential neurotherapeutic and diagnostic applications. Rh‐AMs were successfully synthesized and validated using NMR, FTIR, UV–vis, and fluorescent spectroscopy. The ratio of labeled to unlabeled AMs necessary for Rh‐AM‐NPs formation was optimized via flash nanoprecipitation to confirm the minimum quantity required for direct visualization within cells. Using an in vitro BV2 microglial model, we demonstrated that Rh‐AM‐NPs exhibit multifunctional properties, suppressing the microglial inflammatory response and reducing microglial uptake of fAβ within a low‐toxicity range, while simultaneously enabling in situ tracking of cellular interactions. This work validates a novel nanoplatform for combined AD therapy and diagnostics.

## Graphical abstract

This study presents rhodamine B‐tagged (Rh) AMs (Rh‐AMs) that create stable nanoparticles (Rh‐AM‐NPs) with potential neurotherapeutic and diagnostic applications. Using an in vitro BV2 microglial model, Rh‐AM‐NPs exhibit multifunctional properties, suppressing the microglial inflammatory response, reducing microglial uptake of fAβ within a low‐toxicity range, and enabling in situ tracking of cellular interactions. This work validates a novel nanoplatform for combined AD therapy and diagnostics.

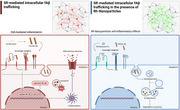

## 1 Introduction

Alzheimer's disease (AD) is the most common form of dementia and is a progressive neurodegenerative disorder [1]. It is characterized by the accumulation of amyloid‐beta (Aβ) plaques, tau pathology, and chronic neuroinflammation. Despite extensive research, developing disease‐modifying therapies remains a significant challenge, primarily due to the complex interactions between pathogenic protein aggregation, impaired clearance mechanisms, and prolonged microglial activation, the brain's resident immune cells. Dysfunction in microglial function not only worsens Aβ accumulation but also leads to synaptic loss and neuronal damage through the release of pro‐inflammatory cytokines and oxidative species. Therefore, strategies that can modulate microglial behavior and enhance Aβ clearance hold significant promise for therapeutic interventions [2, 3, 4, 5, 6].

Recently, we reported the synthesis of amphiphilic macromolecules (AMs) via esterification of sugar‐based diacids, specifically mucic acid and tartaric acid. These sugars were conjugated with hydrophobic aliphatic chains and then with hydrophilic poly (ethylene glycol) (PEG), resulting in architectures that exhibit biological stability and receptor affinity. Our AM nanoparticles (AM–NPs) blocked Aβ fibrillization, reduced the uptake of prefibrillar Aβ, decreased inflammation, enhanced degradation, and protected against neurotoxicity [7, 8, 9]. One of the major barriers to effective treatment is the limited ability of most therapeutic agents to cross the blood‐brain barrier (BBB) and access brain tissue. Additionally, many current approaches lack specificity in targeting pathological processes within defined cellular compartments. Our nanotechnology‐based drug delivery platforms offer a promising solution to overcome these limitations by enhancing brain penetration, improving cellular targeting, and enabling real‐time imaging of therapeutic distribution [9, 10]. In this manuscript, the term ‘theranostic’ is used to describe a cellular‐level, proof‐of‐concept platform that integrates therapeutic activity with fluorescence‐based intracellular visualization.

To facilitate real‐time cellular tracking and imaging, we covalently attached a rhodamine‐B fluorophore to the AM polymer backbone using carbodiimide‐mediated coupling chemistry. This fluorescent tag allows for direct visualization of polymer uptake, intracellular distribution, and colocalization with amyloid aggregates and lysosomal markers in microglial cells. The rhodamine‐B‐labeled AMs were further formulated into nanoparticles (Rh‐AM‐NPs) via flash nanoprecipitation, yielding stable, monodisperse nanostructures with controlled size and surface properties. In this study, we present a translational nanotechnology platform based on AMs engineered to interact with microglial scavenger receptors that are involved in Aβ uptake and degradation. Our optimized nanoparticle formulations include minimal Rh‐AM content, requisite for imaging but low enough to avoid potential cytotoxicity or destabilization. The Rh‐labeled nanoparticles (Rh‐AM‐NPs) were then used to track cellular uptake and intracellular dispersion in microglial models.

**FIGURE 1:**
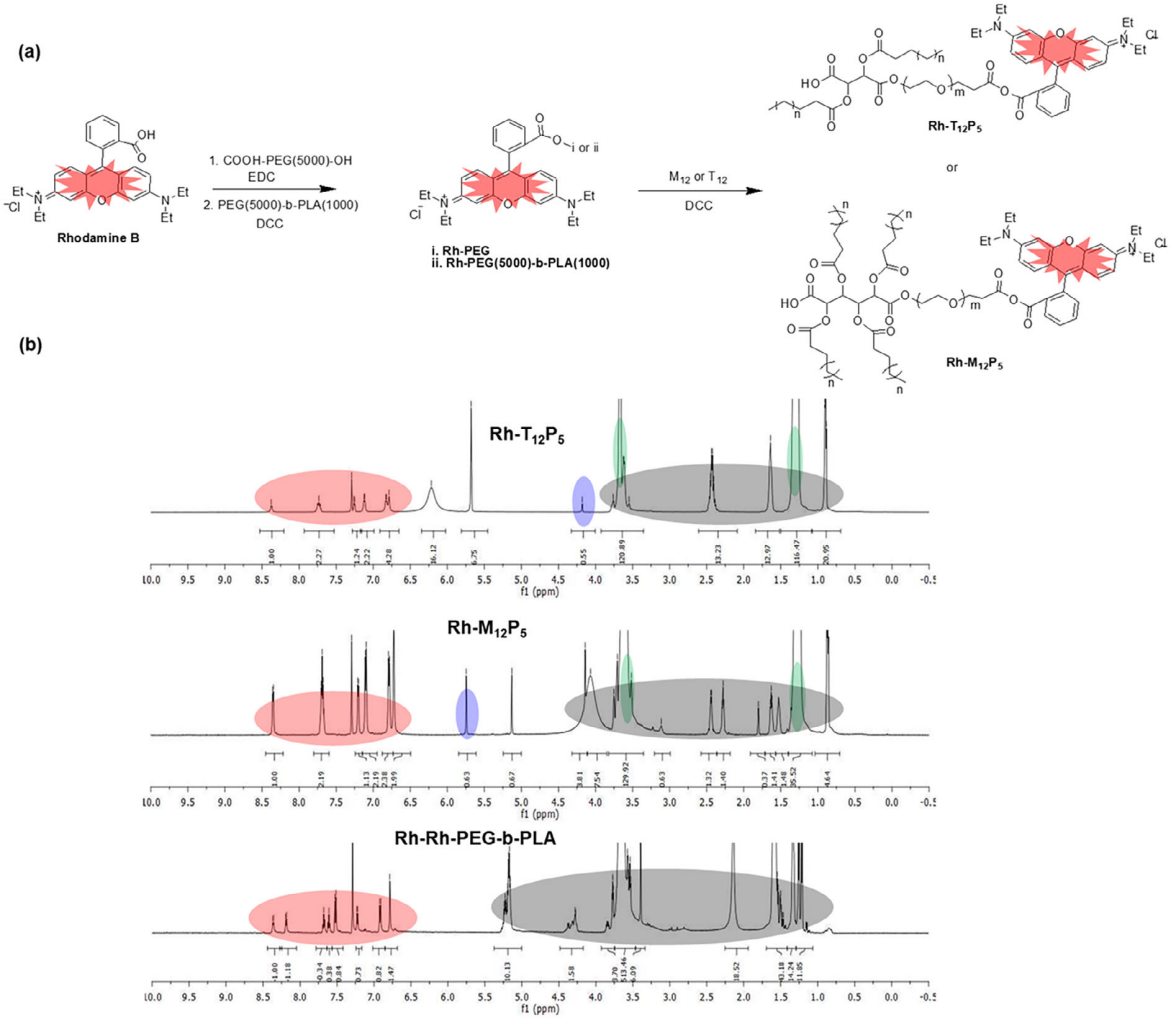
(a) Synthetic approach for Rhodamine‐tagged amphiphilic molecules Rh‐X_12_P_5_ (X = T, M) and their precursors Rh‐X_12_ via esterification reactions (b) structure confirmation by ^1^H NMR: Rh‐T_12_P_5_ (top), Rh‐M_12_P_5_ (middle), and Rh‐PEG‐b‐PLA (bottom) in CDCl_3_.

Our results demonstrate the feasibility of combining targeted nano‐delivery with fluorescence‐based imaging to explore cellular mechanisms of Aβ processing. This platform represents a significant step toward the rational design of microglia‐targeted therapeutics, with the potential to modify the disease course in Alzheimer's disease. Parameters that facilitate nanoparticle permeability across the BBB, such as endothelial cell receptor specificity, charge, and size, need to be considered in nanoparticle design. Specifically, we synthesized fluorescently labeled Rh‐tagged AM‐NPs as a cellular‐level theranostic prototype to enhance the clearance of prefibrillar Aβ through precise targeting of scavenger receptors and modulation of the microglial phenotype (Figure 1).

## 2 Material and Methods

### 2.1 Rh‐AMs Synthesis

All reagents and solvents were obtained from Sigma–Aldrich, TCI Chemicals, or Fisher Scientific, unless otherwise noted, and were used without further purification. Mucic acid, tartaric acid, lauroyl chloride, poly (ethylene glycol) (PEG, Mw = 5000 Da), and rhodamine B were utilized as precursors for the synthesis of amphiphilic macromolecules (AMs). Carbodiimide coupling reagents, including EDC, DCC, NHS, and DPTS, were employed for the conjugation reactions. The macromolecular shells M_12_P_5_ and T_12_P_5_ were synthesized via esterification reactions conducted in two steps, as previously described [8, 11, 12].

### 2.2 Rh‐PEG (1)

A round‐bottom flask containing a stir bar was flame‐dried under vacuum and then filled with argon. NHS (0.12 mmol, 14 mg), EDC (0.12 mmol, 23 mg), and OH‐PEG‐COOH with a Mw of 5000 g/mol (0.1 mmol, 500 mg) were solubilized in 3 mL of dry CH_2_C_l2_. From a second reaction flask, also flame‐dried and filled with argon, the solution of DMAP (0.05 mmol, 6 mg) and Rhodamine B (0.1 mmol, 48 mg) in 2 mL of dry CH_2_C_l2_ was added dropwise into the first flask. After 24 hr of reflux under an argon atmosphere, the obtained mixture was washed with 10 mL of 0.5 N HCl, a saturated NaHCO_3_ solution, and a saturated NaCl solution. The organic phase was dried with anhydrous Na_2_SO_4,_ and then the solvent was evaporated. The red crude product was purified by flash column chromatography using a 1:9 mixture of MeOH/CH_2_Cl_2_. The pure product was collected by evaporation of the solvent under reduced pressure (yield = 61%). ^1^H NMR (600 MHz, CDCl_3_): δ 8.06 (d, 2H), 7.64 (d, 4H), 7.23 (s, 2H), 6.65 (d, 4H), 6.49 (s, 2H), 6.40 (d, 2H), 5.32 (s, 4H), 4.18 (d, 2H), 3.54 (m, ∼4kH), 2.69 (s, 4H), 2.34 (s, 2H), 2.23 (s, 2H), 1.20 (m, 64 H), and 0.90 (m, ∼12H). IR (cm^−1^): 1087 (C─O), 1749 (C═O), 2875 (C─H). UV–vis: 566 nm.

### 2.3 Rh‐PEG‐b‐PLA (2)

PEG (5000)‐b‐PLA (1000) (3.0 g, 1.0 mmol), Rhodamine B (580 mg, 1.1 mmol), and DPTS (0.18 g, 1.0 mmol) in methylene chloride (30 mL) were added at room temperature. DMF (3.0 mL) was also added to ensure a clear, homogeneous solution. After 10 min under nitrogen, 2.0 mL of DCC solution (1.0 M in methylene chloride) was added dropwise over 15 min. After 24 hr, the reaction mixture was washed with 20 mL portions of brine (5×), dried over anhydrous sodium sulfate, and evaporated to dryness. The crude product was purified by precipitation into diethyl ether (100 mL) from methylene chloride (5 mL). Product was obtained as a red, waxy solid (2.6 g, 82% yield). ^1^H NMR (CDCl_3_) (δ): 8.79 (d, 1H), 8.19 (d, 2H), 7.68 (m, 1H), 7.61 (m, 1H), 7.52 (m, 2H), 7.23 (d, 1H), 6.92 (d, 2H), 6.79 (s, 2H), 5.20 (m, 4H), 4.28 (m, 2H), 3.66 (m, ∼4kH), 3.39 (s, 8H), 2.14 (s, 12H), 1.55 (m, 64H), 1.34 (s, 8H), and 1.21 (m, 12H). ^13^C NMR (CDCl_3_, 600 MHz): 170.02, 169.56, 169.31.169.11, 157.80, 155.39, 132.18, 131.94, 131.71, 130.18, 129.52, 113.96, 113.52, 96.28, 71.88, 70.51, 69.14, 68.95, 68.74, 66.58, 66.53, 64.40, 59.00, 46.16, 31.53, 22.60, 20.48, 20.08, 16.71, 16.62, 12.76. IR (cm^−1^): 1096 (C─O), 1750 (C═O), 2882 (C─H). UV–vis: 568 nm. T_d_ = 366.6°C (peak value by TGA); T_g_ = 51.8°C (peak value by DSC).

### 2.4 T_12_ and M_12_

Mucic acid or tartaric acid (4.2 g, 20 mmol) and zinc chloride (0.28 g, 2.0 mmol) were added to lauroyl chloride (37 mL, 160 mmol). The reaction mixture was heated to 90°C for 10–12 hr. After cooling to room temperature, diethyl ether (20 mL) was added to the reaction mixture, and the mixture was stirred into ice water (∼150 mL). Additional diethyl ether (80 mL) was added to the mixture, and stirring was continued for an additional 30 min. The ether portion was separated, washed with brine to neutral pH (∼7), dried over anhydrous sodium sulfate, and evaporated to dryness. The crude product was purified by precipitation into petroleum ether (200 mL) from diethyl ether (20 mL). The product was obtained as a white powder (13 g, 68% yield).

### 2.5 Rh‐T_12_P_5_

Rh‐PEG (4.0 g, 1.0 mmol), T_12_ (2.2 g, 3.0 mmol), and DPTS (0.2 g, 1.0 mmol) in methylene chloride (30 mL) were added at room temperature. DMF (3.0 mL) was also added to ensure a clear, homogeneous solution. After 10 min under nitrogen, 3.0 mL of DCC solution (1.0 M in methylene chloride) was added dropwise over 15 min. After 48 hr, the reaction mixture was washed with 20 mL portions of brine (5×), dried over anhydrous sodium sulfate, and evaporated to dryness. The crude product was purified by precipitation into diethyl ether (100 mL) from methylene chloride (5 mL). Product was obtained as a red, waxy solid (3.7 g, 85% yield). ^1^H NMR (CDCl_3_) (δ): 8.38 (s, 1H), 7.73 (m, 2H), 7.25 (m, 1H), 7.13 (m, 2H), 6.82 (m, 4H), 7.22 (br s, 1H), 5.68 (s, 4H), 4.18 (d, 2H), 3.67 (m, ∼4kH), 2.43 (m, 12H), 1.64 (s, 12H), 1.30 (m, ∼64H), and 0.90 (m, ∼12H). ^13^C NMR (CDCl_3_, 600 MHz): 166.86, 157.84, 155.40, 131.97, 131.79, 130.17, 129.42, 113.92, 96.18, 76.92, 72.61, 71.92, 71.26, 70.55, 70.27, 68.48, 65.01, 59.05, 46.12, 33.75, 31.91, 29.62, 29.47, 29.02, 25.76, 22.68, 14.14, 12.72. IR (cm^−1^): 1093 (C─O), 1746 (C═O), 2872 (C─H). UV–vis: 560 nm. T_d_ = 390.3°C (peak value by TGA); T_g_ = 49.9°C (peak value by DSC).

### 2.6 Rh‐M_12_P_5_

Rh‐PEG (4.0 g, 1.0 mmol), M_12_ (3 g, 3.0 mmol), and DPTS (0.3 g, 1.0 mmol) in methylene chloride (30 mL) were added at room temperature. DMF (3.0 mL) was also added to ensure a clear, homogeneous solution. After 10 min under nitrogen, 3.4 mL of DCC solution (1.0 m in methylene chloride) was added dropwise over 15 min. After 48 hr, the reaction mixture was washed with 20 mL portions of brine (5×), dried over anhydrous sodium sulfate, and evaporated to dryness. The crude product was purified by precipitation into diethyl ether (100 mL) from methylene chloride (5 mL). Product was obtained as a red, waxy solid (3.0 g, 76% yield). ^1^H NMR (CDCl_3_) (δ): 8.36 (d, 1H), 7.70 (m, 2H), 7.29 (d, 1H), 7.21 (m, 2H), 6.79 (m, 2H), 6.72 (s, 2H), 5.74 (s, 4H), 5.13 (s, 2H), 4.07 (m, ∼2H), 3.64 (m, ∼4kH), 2.45 (m, 6H), 2.28 (m, 6H), 1.80 (s, 4H), 1.64 (m, 6H), 1.62 (s, 6H), 1.31 (m, ∼64H), and 0.87 (m, ∼12H). ^13^C NMR (CDCl_3_, 600 MHz): 172.87, 172.69, 171.74, 166.93, 157.82, 155.38, 131.52, 132.15, 131.84, 131.67, 130.16, 129.46, 113.85, 96.14, 72.57, 71.88, 70.52, 69.24, 68.65, 67.88, 64.69, 61.60, 59.01, 49.00, 46.08, 33.49, 31.88, 29.61, 29.49, 29.33, 25.56, 24.87, 22. 65, 14.12, 12.67. IR (cm^−1^): 1090 (C─O), 1743 (C═O), 2885 (C─H). UV–vis: 570 nm. T_d_ = 389.8°C (peak value by TGA); T_g_ = 44.1°C (peak value by DSC).

### 2.7 Rh‐AMs Characterization

Nuclear magnetic resonance (^1^H and ^13^C NMR) spectra of the products were obtained using a Bruker Avance NEO 60000 MHz spectrophotometer. Samples were dissolved in chloroform‐d. Fourier transform infrared (FT‐IR) spectra were recorded on a Thermo iS 10 FT‐IR spectrometer using OMNI software as an average of 32 scans. UV–vis absorption spectroscopic data were obtained using a UV−vis spectrophotometer (HP 8453). Melting temperatures were determined by DSC: DSC: Netzsch DSC 214 Polyma and TGA: Netzsch TG 209 F1 Libra.

### 2.8 Nanoparticle Formulation via Flash Nanoprecipitation

Rhodamine‐labeled nanoparticles (Rh‐AM‐NPs) were synthesized using a flash nanoprecipitation (FNP) technique via a confined impinging jet (CIJ) mixer, as previously described [7, 12, 13, 14]. Briefly, the organic phase was prepared by first dissolving 6 mg/mL of the original amphiphilic shell molecule in 125 µL tetrahydrofuran (THF; Sigma–Aldrich, St. Louis, MO). This solution was then combined with a 2 mg/mL rhodamine‐conjugated shell molecule to yield a final 250 µL organic stream containing 8 mg/mL total shell (6 mg/mL un‐tagged AMs + 2 mg/mL rhodamine‐tagged) and 2.5 mg/mL of a hydrophobic core molecule. This organic stream was impinged against 250 µL of an aqueous stream using the CIJ mixer, with the mixing time (Tmix) controlled to ensure that the nanoparticle formation time (Tflash) exceeded Tmix, thereby promoting a uniform nanoparticle size distribution. The resulting nanoparticle suspension was immediately diluted into a 9‐fold volume of ultrapure water to arrest further aggregation and enhance stabilization. The nanoparticles were then dialyzed against water using a 3.5 kDa MWCO dialysis cassette (Thermo Fisher Scientific, Waltham, MA) to remove residual solvents and unincorporated materials. Dynamic light scattering (DLS) was performed using a Malvern Zetasizer Nano ZS90 to determine the hydrodynamic radius and polydispersity index of the Rh‐NPs. Parameters such as size, surface charge, and critical concentration for the base nanoparticle formulation have been previously characterized [12, 13, 15].

## 3 Nanoparticle Characterization

### 3.1 Dynamic Light Scattering (DLS) and Zeta Potential Measurements

Dynamic light scattering (DLS), zeta potential, and polydispersity index (PDI) were measured using a NanoZS90 instrument from Malvern Instruments in Southboro, MA. Prior to measurement, samples were dissolved in deionized (DI) water. Each sample was analyzed in triplicate, with 30 measurements per run at 25°C. The results for particle sizes are presented as the mean ± standard deviation based on intensity distribution.

### 3.2 Transmission Electron Microscopy (TEM)

For nanoparticle imaging, NPs were loaded onto a formvar‐coated, carbon‐stabilized copper grid with a mesh size of 400 (Pacific Grid‐tech). Excess solution was removed using Whatman filter paper, after which the nanoparticles were imaged directly. The images were captured at a magnification of 22 000 at a voltage of 80 kV.

### 3.3 Fluorescence Spectroscopy

The fluorescence spectra of Rh‐NPs were measured on a Horiba PTI QM‐400 Fluorescence spectrophotometer at an excitation wavelength of 530 nm, and the slit widths of excitation and emission were all fixed at 1.5 nm. Emission spectra were recorded from 535 to 750 nm.

### 3.4 Preparation and Characterization of Aβ1–42 Fibrils

Aβ1–42 fibrils were prepared as our previously reported protocol [9, 16]. Briefly, Aβ1–42 peptide (Anaspec, Fremont, CA) was first dissolved in 100% 1,1,1,3,3,3‐hexafluoro‐2‐propanol (HFIP) to a final concentration of 5 mg/mL, aliquoted, and then air‐dried overnight at room temperature in a fume hood. The dried peptide was reconstituted in DMSO to a final concentration of 5 mM and sonicated for 1 min in low‐binding microcentrifuge tubes (Corning, Manassas, VA). The peptide solution was then diluted to 200 µm in phosphate‐buffered saline (PBS) containing 0.2% sodium dodecyl sulfate (SDS) and incubated at 37 °C for a minimum of 4 weeks with constant shaking at 300 rpm to promote fibril formation. Fibrils were collected by centrifugation at 5000 × g for 1 hr at 4 °C. The concentration of Aβ fibrils was determined by measuring absorbance at 280 nm, using an extinction coefficient of 1280 M^−^^1^ cm^−^^1^ [10]. Fibril formation was confirmed by thioflavin‐T (Th‐T) fluorescence assay and transmission electron microscopy (TEM).

## 4 Cell Culture

### 4.1 BV2 Mouse Microglia Cell Line

BV2 microglial cells were generously provided by Dr. Bin Liu (University of Florida) and Dr. Jason Richardson (Northeast Ohio Medical University). Cells were maintained in Dulbecco's Modified Eagle Medium (DMEM; Gibco, Manassas, VA) supplemented with 10% fetal bovine serum (FBS) and 1% penicillin–streptomycin (Pen/Strep; Gibco). For experiments, BV2 cells were seeded at a density of 20,000 cells per well in 96‐well plates and allowed to adhere for 24 hr. All subsequent treatments were carried out in DMEM supplemented with 1% FBS and 1% Pen/Strep to minimize basal activation while maintaining cell viability.

### 4.2 Cytotoxicity Assays

To assess the cytocompatibility of Rh‐NPs, a cytotoxicity assay was performed by measuring lactate dehydrogenase (LDH) release using the Promega non‐radioactive cytotoxicity kit. Briefly, BV2 microglial cells were plated at 20,000 cells per well in a 96‐well plate and were treated with serial dilutions of Rh‐NPs with varying compositions of Rh‐AMs (0.5, 1, 2 mg) for 24 h, After which 50 µL of the media was collected. The level of LDH released was then measured by assessing the absorbance of the red formazan product at 490 nm using a Tecan Infinite M200 Pro microplate reader. Data were normalized to the maximum LDH release control

### 4.3 BV2 Microglia Activation and Cytokine Assays

BV2 cells were seeded at a density of 20 000 cells per well in a 96‐well plate and allowed to adhere overnight. Cells were then co‐treated with 20 µM fAβ in the presence or absence of nanoparticles (NPs) or with 10 ng/mL lipopolysaccharide (LPS) as a positive control. After 24 hr of treatment, culture supernatants were collected, and nitric oxide (NO) production was measured using the Griess reagent assay (Promega, Madison, WI). Following supernatant collection, cells were fixed with 4% paraformaldehyde (PFA), washed with phosphate‐buffered saline (PBS), and processed for immunocytochemical assays.

### 4.4 Immunocytochemistry

Following plating, cells were fixed with 4% PFA, washed with PBS, and permeabilized by adding PBS‐T for 10 min at room temperature. The cells were then washed again with PBS before incubation for 1 hr with 2% Goat Blocking Buffer to reduce non‐specific antibody binding and background noise. Cells were then incubated overnight at 4°C with a primary anti‐iNOS antibody (1:200, Invitrogen). The following day, the cells were subjected to a second permeabilization step and then incubated for 1 hr at room temperature with the Alexa 488‐conjugated secondary antibody (1:200, Invitrogen). Cells were also counterstained with Hoechst before being imaged using the Keyence BZ‐X810 microscope.

## 5 Results

### 5.1 Synthesis and Physicochemical Characterization of Rhodamine‐B Tagged AM–NPs

The amphiphilic macromolecules (AMs), Rh‐T_12_P_5_ and Rh‐M_12_P_5_, were successfully synthesized through the esterification and PEGylation of sugar‐based diacids (tartaric acid or mucic acid), followed by rhodamine‐B conjugation using carbodiimide‐mediated coupling. The successful incorporation of rhodamine‐B was confirmed through ^1^H NMR (Figure 1), IR, UV–vis, and fluorescent spectroscopy, which displayed distinct absorption and emission characteristics suitable for bioimaging applications (see Supporting Information).

**FIGURE 2:**
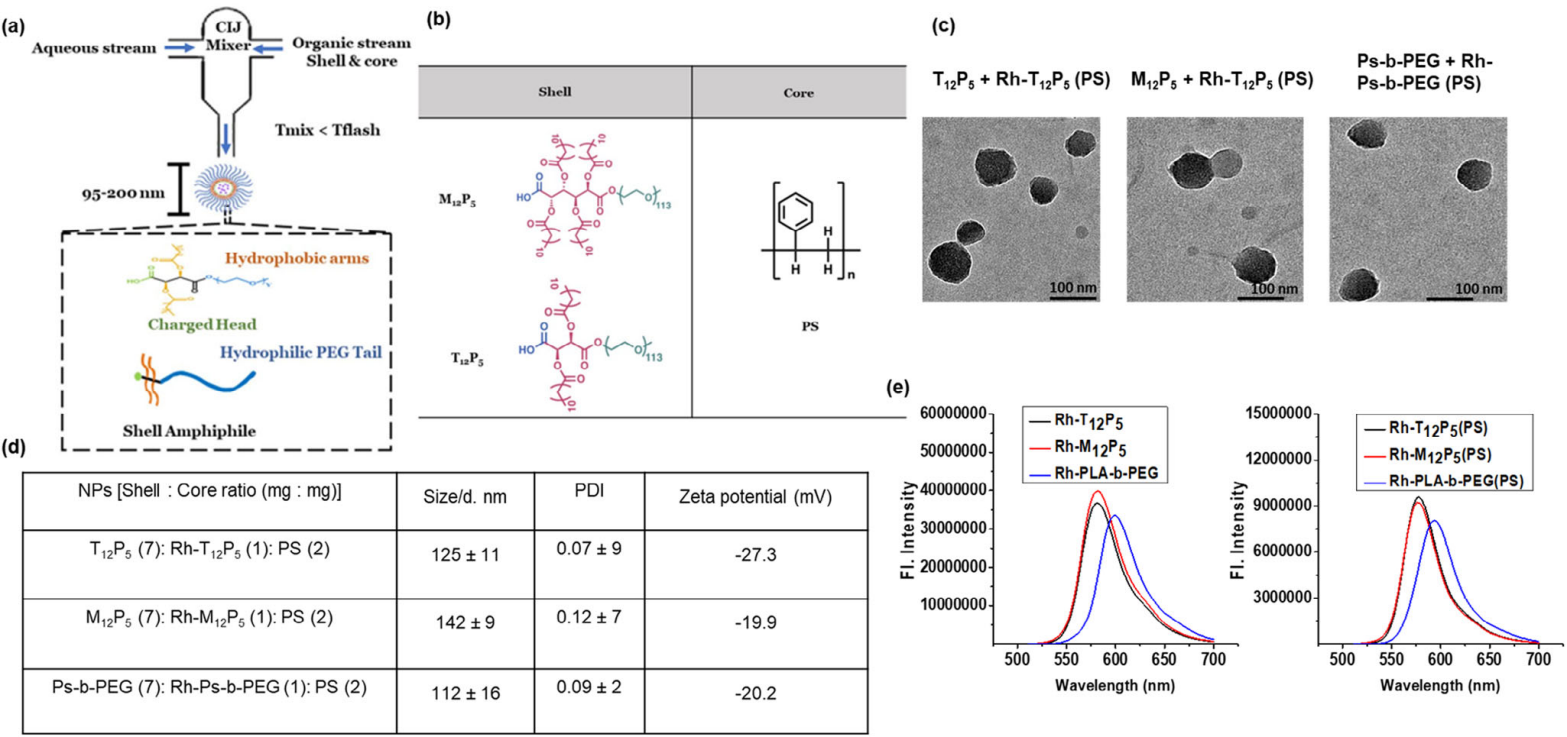
(a) Schematic diagram of nanoparticle (Rh‐AM‐NP) fabrication via flash nanoprecipitation (FNP). (b) Table of the chemical structures of Rh‐AM shells and polystyrene core. (c) TEM images of Rh‐AM‐NPs. (d) Table summarizing the hydrodynamic characterization of Rh‐AM‐NPs. Data are presented as mean ± SEM; *n* = 3 (e) Fluorescent spectrum Rh‐AMs and Rh‐AM‐NPs. The excitation wavelength of 530 nm, and the slit widths of excitation and emission were all fixed at 1.5 nm. Emission spectra were recorded from 535 to 750 nm.

Self‐assembly of the rhodamine‐labeled AMs into nanoparticles (Rh‐AM–NPs) was achieved via flash nanoprecipitation (FNP), yielding stable, monodisperse nanostructures. Dynamic light scattering (DLS) measurements revealed hydrodynamic diameters of 125 ± 11 nm for Rh‐T_12_P_5_‐NPs, 142 ± 9 nm for Rh‐M_12_P_5_‐NPs, and 112 ± 16 nm for Rh‐PEG‐b‐PLA NPs (Figure 2a,b). All formulations exhibited low polydispersity indices (PDI < 0.12), indicating uniform particle populations, and maintained negative zeta potentials (−19.9 to −27.3 mV), suggesting good colloidal stability.

Transmission electron microscopy (TEM) confirmed the spherical morphology of the nanoparticles (Figure 2c), while fluorescence spectroscopy demonstrated robust rhodamine emission across all Rh‐AM–NP formulations (Figure 2e). The fluorescent spectra for both Rh‐AMs and Rh‐AM‐NPs, including their excitation and emission spectra, were recorded from 535 to 750 nm. Collectively, these findings affirm the effective formulation of trackable Rh‐AM‐NPs with desirable physicochemical properties for biological applications.

### 5.2 Rh‐AM–NPs Cytotoxicity and Cellular Uptake

**FIGURE 3:**
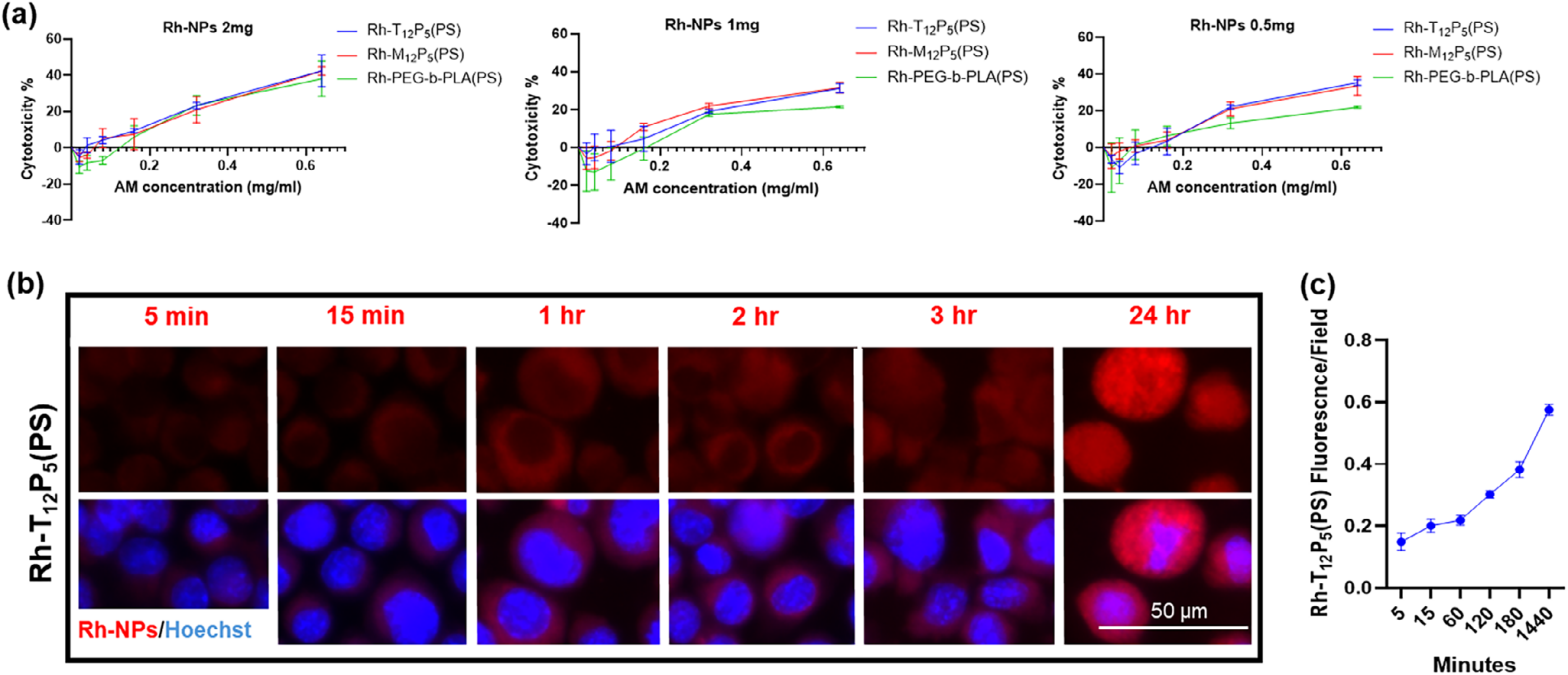
Rh‐AM–NPs cytotoxicity and cellular uptake. (a). Cytotoxicity assessment of Rh‐AM‐NPs via lactate dehydrogenase cellular release. BV2 microglial cells were treated with serial dilutions of Rh‐AM‐NPs (Rh‐T_12_P_5_(PS), Rh‐M_12_P_5_(PS), Rh‐PEG‐b‐PLA(PS) with varying compositions of Rh‐AMs (0.5, 1, 2 mg) for 24 h. The level of LDH released was then measured in the collected media. Data were normalized to the maximum LDH release control. Data are from *n* = 3 and means ± SEM. (b,c). Kinetics of Rh‐AM‐NPs (Rh‐T_12_P_5_(PS)) cellular uptake. BV2 cells were incubated with Rh‐NPs and then fixed with 4% PFA at various time points (5, 15, 60, 120, 180, and 1440 min). Intracellular Rh‐AM‐NPs were visualized in counterstained cells using fluorescence microscopy. (c). The fluorescence of internalized Rh‐AM‐NPs was quantified at different time points, demonstrating linear accumulation within BV2 cells for up to 24 h.

To assess the cytotoxic range and biocompatibility of the newly developed Rh‐AM‐NPs, considering the reported level of rhodamine toxicity at high concentrations [17], the cytotoxicity effects of Rh‐AM‐NPs were evaluated using BV2 cells. BV2 cells were incubated for 24 hr with serial dilutions of Rh‐AM‐NPs containing different amounts of Rh‐AM (1, 0.5, and 0.1 mg) (Figure 3a) fabricated within the Rh‐AM‐NPs. The level of LDH released from BV2 cells was then measured and normalized to the maximum LDH release control. All Rh‐AM‐NPs exhibited a low cytotoxicity range (<20%), except those with a high content of Rh‐AMs and a high concentration range (0.16 and 0.08 mg/mL). Interestingly, Rh‐AM‐NPs at the high concentration ranges (0.16 and 0.08 mg/ml) didn't exceed 40% of cell toxicity (IC50 is 0.78, 0.769, and 0.801 mg/ml for T_12_P_5_(PS), M_12_P_5_(PS), and PEG‐b‐PLA(PS), respectively) (Figure 3a).

Next, we investigated the ability of Rh‐AM‐NPs to be internalized by BV2 cells in real time. To test this, BV2 cells were incubated with Rh‐AM‐NPs and then fixed with 4% PFA at different time points (5, 15, 60, 120, 180, and 1440 min). Then, cells were counterstained with Hoechst and imaged at 564 nm to visualize intracellular Rh‐AM‐NPs (Figure 3b; Figures S1 and S2). The three Rh‐AM‐NPs exhibited robust, gradual internalization within cells over 24 hr (Figure S3), with Rh‐T_12_P_5_(PS) showing the fastest saturation in BV2 cells, especially at earlier time points (Figure 3b; Figure S3).

### 5.3 Rh‐AM‐NPs Interrupt fAβ Internalization and Modulate Microglial Inflammatory Response

**FIGURE 4:**
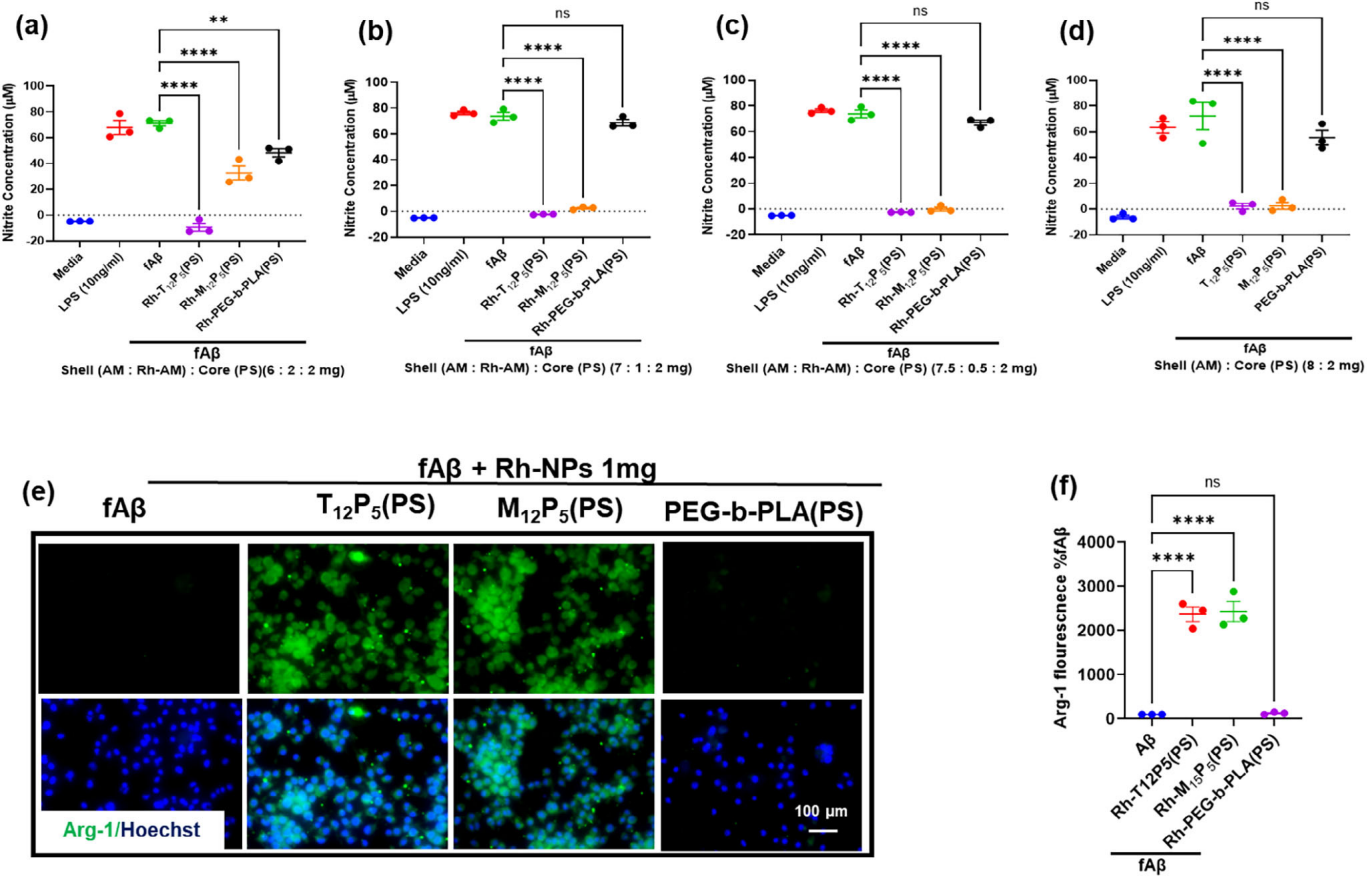
Rh‐AM‐NPs suppress fAβ‐mediated inflammatory response in BV2 cells. BV2 microglial cells were co‐treated with 20 µm fAβ in the presence or absence of Rh‐AM‐NPs with varying compositions of Rh‐AMs (0.5, 1, 2 mg) for 24 h. Additionally, a positive control was modeled by incubating BV2 microglia with 10 ng/ml LPS. (a,d). Conditioned media were collected from the three Rh‐NP sets (a–c) or unlabeled NPs (d) and assayed for nitrite content using the Griess assay. (e). Fluorescence images of Arg‐1 (green) immunoreactivity in BV2 cells treated with an intermediate amount (1 mg) of Rh‐NPs. (f). Quantitative analysis of Arg‐1 expression in BV2 cells treated with fAβ in the presence or absence of Rh‐NPs (1 mg). Data are mean ± SEM. Data analysis was conducted using one‐way ANOVA followed by Dunnett's multiple comparisons test.

**FIGURE 5:**
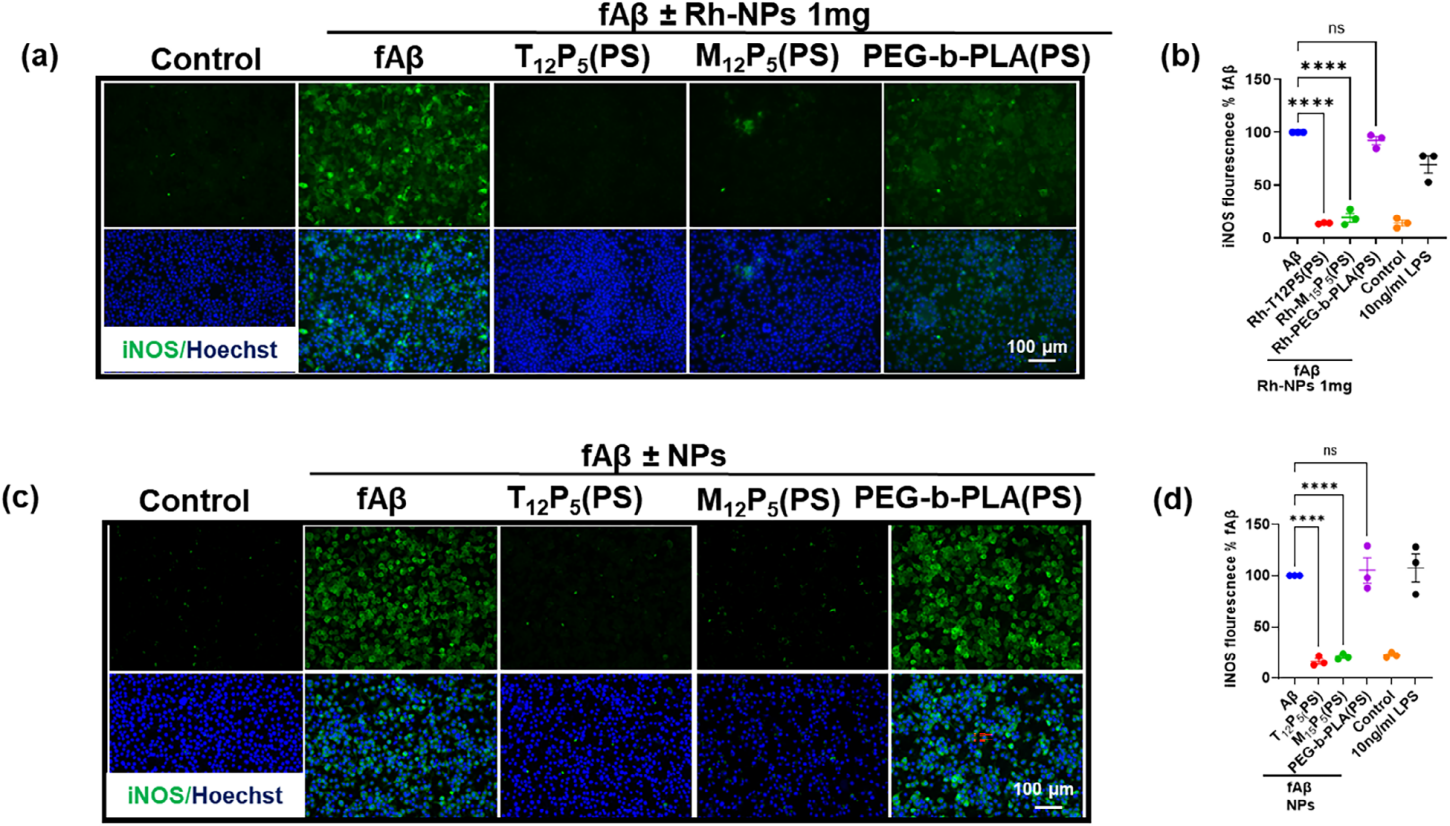
Rh‐NPs suppress fAβ‐induced iNOS expression in BV2 cells. BV2 microglial cells were co‐treated with 20 µM fAβ in the presence or absence of Rh‐NPs with varying compositions of Rh‐AMs (0.5, 1, 2 mg) for 24 h. Additionally, a positive control was modeled by incubating BV2 microglia with 10 ng/mL LPS. Fluorescence images of iNOS (green) immunoreactivity in BV2 cells treated with an intermediate amount (1 mg) of Rh‐NPs (a) or unlabelled NPs (c). (b–d). Quantitative analysis of iNOS expression in BV2 cells treated with fAβ in the presence or absence of Rh‐NPs (1 mg). Data are mean ± SEM. Data analysis was conducted using one‐way ANOVA followed by Dunnett's multiple comparisons test.

Alzheimer's disease is associated with pro‐inflammatory microglial activation and phenotypic shifts in microglia. To evaluate the ability of Rh‐AM‐NPs to modulate the fAβ‐induced inflammatory response in microglia, we measured nitrite release and the microglial expression of inducible nitric oxide synthase (iNOS), which directly reflects the activation of the iNOS‐mediated inflammatory pathway. BV2 cells were co‐treated with 20 µM fAβ in the presence or absence of Rh‐AM‐NPs at varying Rh‐AM amounts (0.5, 1, and 2 mg), or with 10 ng/mL lipopolysaccharide (LPS) as a positive control. At lower Rh‐AM amount (0.5 and 1 mg), both Rh‐T_12_P_5_(PS) and Rh‐M_12_P_5_(PS) significantly reduced nitrite release (Figure 4a–c; Figure S1) and iNOS expression in BV2 cells compared to fAβ‐only controls (Figure 5a,b). Similarly, the unlabeled NPs‐treated cells showed no detectable nitrite release and no iNOS expression (Figures 4d and  5c,d). However, at the highest Rh‐AM content tested (2 mg), Rh‐M_12_P_5_(PS) failed to suppress inflammation, as evidenced by significantly elevated iNOS expression (Figure S1). Notably, Rh‐T_12_P_5_(PS) maintained its anti‐inflammatory effect even at the highest Rh‐AM content (2 mg), with iNOS expression reduced to levels comparable to those in the vehicle‐only control, regardless of dosage (Figure S1). In contrast, control Rh‐PEG‐b‐PLA (PS) did not exhibit a significant reduction in nitrite release and iNOS expression at any concentration tested (Figures 4a–c and S1). In general, rhodamine‐tagged AMs are sterically bulkier than tartaric acid or mucic acid‐based AMs. This added bulk may affect nanoparticle packing and shell organization, potentially impacting the more stereochemically complex mucic acid‐derived AMs, and could explain the differences in biological activity at higher rhodamine content in these Rh‐NPs.

**FIGURE 6:**
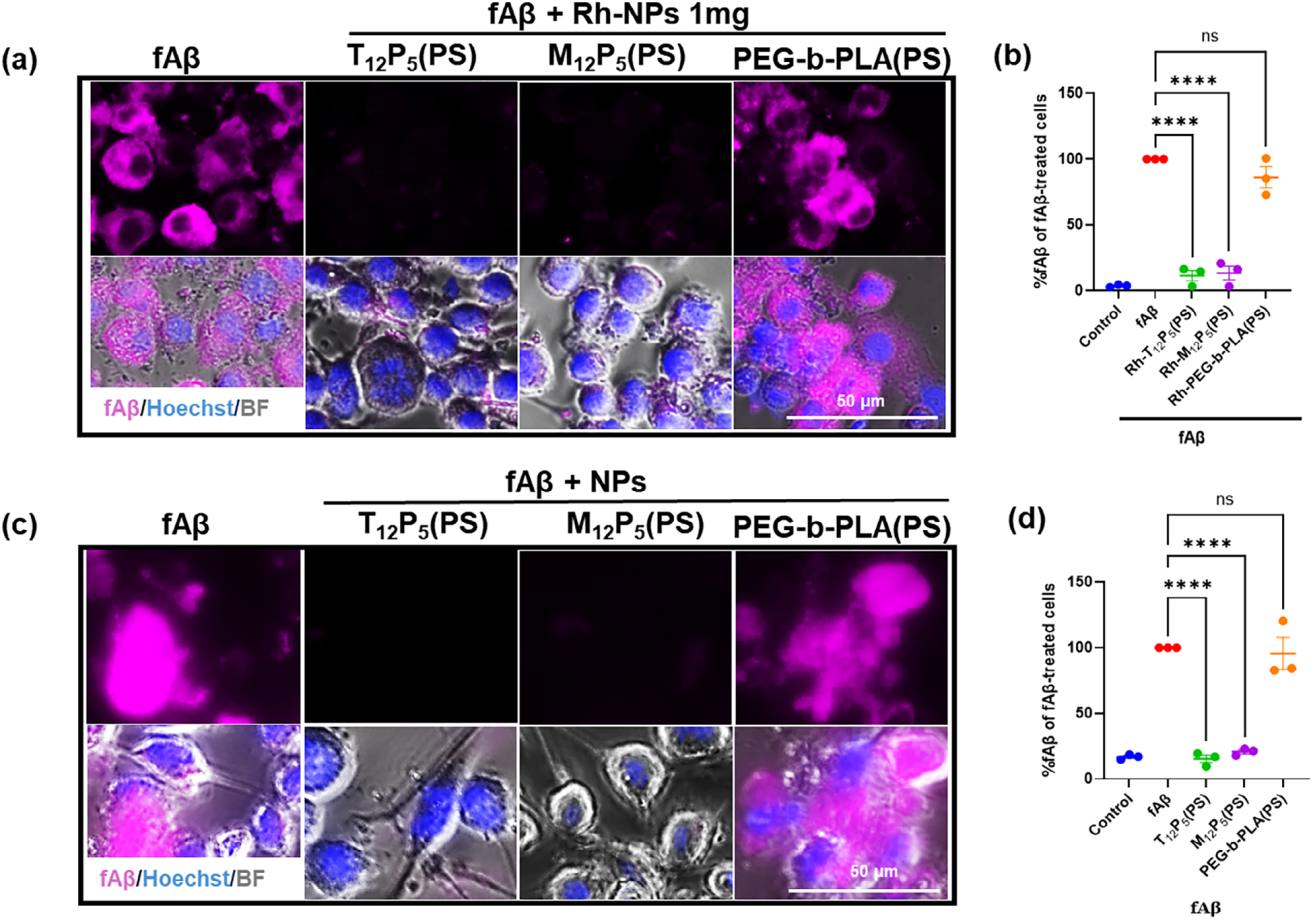
Rh‐NPs interrupt fAβ internalization into BV2 cells. BV2 microglial cells were co‐treated with 20 µm fAβ in the presence or absence of Rh‐NPs containing 1 mg of Rh‐AMs (a,b) or unlabeled‐NPs (c,d) for 24 h. (a,b). Cells were fixed, then, using immunocytochemistry, the expression of iNOS was assayed. (b,c). Quantitative analysis of iNOS in BV2 cells treated with fAβ in the presence or absence of Rh‐NPs (b) or unlabeled‐NPs (d). Data are mean ± SEM. Data analysis was conducted using one‐way ANOVA followed by Dunnett's multiple comparisons test.

Next, given our previous findings demonstrating the efficacy of AMs in inhibiting microglial fAβ internalization [9], we examined the potential effect of Rh conjugation to AMs shells on this function. To test this, we employed the fAβ‐mediated inflammation model of BV2 microglia. BV2 cells have been widely used over the past three decades to recapitulate the inflammatory response observed in primary microglia in neurodegenerative disease [18]. BV2 cells were co‐treated with 20 µM fAβ in the absence or presence of Rh‐AM‐NPs (1 mg of Rh‐AM), a concentration that showed strong anti‐inflammatory activity, minimal toxicity, and contained a high level of Rh‐AMs. The immunoreactivity of intracellular fAβ was measured in Rh‐AM‐NPs‐treated cells and compared with the fAβ‐only‐treated cells (Figure 6a,b). Both Rh‐T_12_P_5_(PS) and Rh‐M_12_P_5_(PS) significantly reduced intracellular fAβ compared to fAβ‐only controls. This reduction in fAβ confirms the bioactive Rh‐AMs' uninterrupted ability to counteract fAβ trafficking. This finding was confirmed by the control Rh‐PEG‐b‐PLA (PS), which did not exert any change on the intracellular fAβ levels compared to fAβ‐only controls (Figure 6a,b). Similarly, the unlabeled NPs‐treated cells showed significantly reduced intracellular fAβ (Figure 6c‐d) compared to cells treated with fAβ and unlabeled control NPs.

## 6 Discussion

In this study, we hypothesized that Rh‐AMs can form stable, functional nanoparticles (Rh‐AM‐NPs) that serve as a theragnostic multimodal approach, offering the potential to trace molecular details of the disease while simultaneously targeting its inflammatory component. Theranostic nanoparticles are generally defined as those that combine therapy with disease‐state sensing. In contrast, Rh‐AM‐NPs act as imaging‐enabled therapeutics and a cellular‐level theranostic prototype, with diagnostic function limited to in vitro fluorescence‐based intracellular tracking. This distinction clarifies their scope while emphasizing the value of cellular imaging for mechanistic studies.

Our previous studies reported SR‐specific AM‐NPs as potent nanotherapeutics that can target multiple aspects of the disease, including extracellular fibril amyloid beta formation and intracellular internalization in BV2 cells [9]. This current study presents a proof‐of‐concept for the efficacy and applicability of the newly developed NPs, which consist of a precise combination of Rh‐labeled and unlabeled AMs.

Several rhodamine fluorophores have been widely used to track the internalization of NPs by tagging the formulation. However, cytotoxic potential varies depending on NPs composition, functionalization, dosage, and cell type. Recently, a study utilizing rhodamine derivative in coordination with gallic acid‐based nanoparticles showed that these NPs were efficiently targeting fAβ‐associated pathologies in vitro and in vivo [19].

Rhodamine‐labeled AMs were used at various concentrations (2, 1, 0.5, and 0.1 mg/mL), with 0.5 mg/mL being utilized for NPs fabrication. A study has reported that the safe concentration for Rhodamine B is considered to be up to 1 mg/mL [20], although there is no FDA‐approved reference to support this claim. Nevertheless, we opted for a concentration of 0.5 mg/mL, which is well within the safe range and far from the toxic levels. These nanoparticles exhibit comparable physical properties and minimal toxicity in BV2 cells, form functional structures that are internalized by microglia, and exert strong anti‐inflammatory effects against fAβ‐induced inflammation. Although the Rh‐NPs demonstrate favorable microglial modulation, their capacity to cross the blood‐brain barrier is a critical parameter that warrants further study in complex physiological models.

## 7 Conclusion

In conclusion, this study reports the development of rhodamine‐labeled amphiphilic macromolecule‐based nanoparticles (Rh‐AM–NPs) as a cellular‐level theranostic prototype for targeted modulation and imaging of microglial responses in Alzheimer's disease. Synthesized from sugar‐based diacids and functionalized with aliphatic chains, PEG arms, and a rhodamine‐B tag, these nanoparticles formed stable, monodisperse structures that interact with microglial scavenger receptors. Characterization revealed favorable size, surface charge, and fluorescence. In vitro assays showed high biocompatibility, specific targeting, and effective cellular uptake. Rh‐AM–NPs significantly reduced Aβ‐induced microglial inflammation and enhanced lysosomal colocalization with Aβ aggregates, highlighting their potential to restore microglial clearance functions.

By combining structural bioactivity with fluorescence imaging, this platform enables both mechanistic studies and therapeutic interventions, positioning Rh‐AM–NPs as a promising tool for addressing neuroinflammation and amyloid pathology in Alzheimer's disease and paving the way for future theranostic strategies in central nervous system disorders.

Future investigations will need to explore the role of Rhodamine‐tagged nanoparticles with specific surface properties in diverting the clearance of fAβ using appropriate in vivo models. This will involve precise targeting of scavenger receptors and the modulation of microglial phenotypes.

## Funding

This work was supported by NIH/National Institute of Aging R21 (1R21AG085394‐01), PI: Hoda M. Gebril, Kathryn E. Uhrich, and Prabhas V. Moghe.

## Conflicts of Interest

All the authors declare no conflicts of interest.

## Supporting information




**Supporting File**: ppsc70082‐sup‐0001‐SuppMat.pdf.

## Data Availability

Data will be made available on request
