# Supplementary material for: Theranostic Nanoparticles for Fluorosensitive Visualization and Inhibition of Amyloid Beta‐Induced Neuroinflammation
Source: Part Part Syst Charact. 2026 Feb 19;43(2):e00220. doi: 10.1002/ppsc.202500220 (PMC13173615; doi:10.1002/ppsc.202500220)
Supplement: Supplementary file 1 — Supporting File: ppsc70082‐sup‐0001‐SuppMat.pdf. [file PPSC-43-e00220-s001.pdf]

*Supporting information to Research Article to Particle and Particle Systems Characterization*

# Theranostic Nanoparticles for Fluorosensitive Visualization and Inhibition of Amyloid Beta-Induced Neuroinflammation

*Umme Tamima[1], Hoda M. Gebril*[2], Md Ragib Hasan[1], Miguel A. Quintero[1], Aravind Aryasomayajula[3], Diya Chengappa[2], Imran Attarwala[2], Bradley L. Truong[1], Prabhas V. Moghe[2,4,5], and Kathryn E. Uhrich*[1]*

[1]Department of Chemistry, University of California, Riverside, CA 92507, USA. [2]Department of Biomedical Engineering, Rutgers University, Piscataway, NJ 08854, USA.

[3]Cooper Medical School, Rowan University, Camden, NJ 08103, USA.

[4] Department of Chemical & Biochemical Engineering, Rutgers University, Piscataway, NJ 08854, USA

[5] University of Texas at Dallas, Richardson, TX 75080, USA

**Corresponding authors:*

*Hoda M. Gebril:* hoda.gebril@rutgers.edu

Department of Biomedical Engineering, 599 Taylor Road, Rutgers University, Piscataway, NJ-08854, USA

*Kathryn E. Uhrich:* kathryn.uhrich@ucr.edu

Department of Chemistry, 900 University Avenue, University of California, Riverside, CA 92507, USA

# Supporting Data:

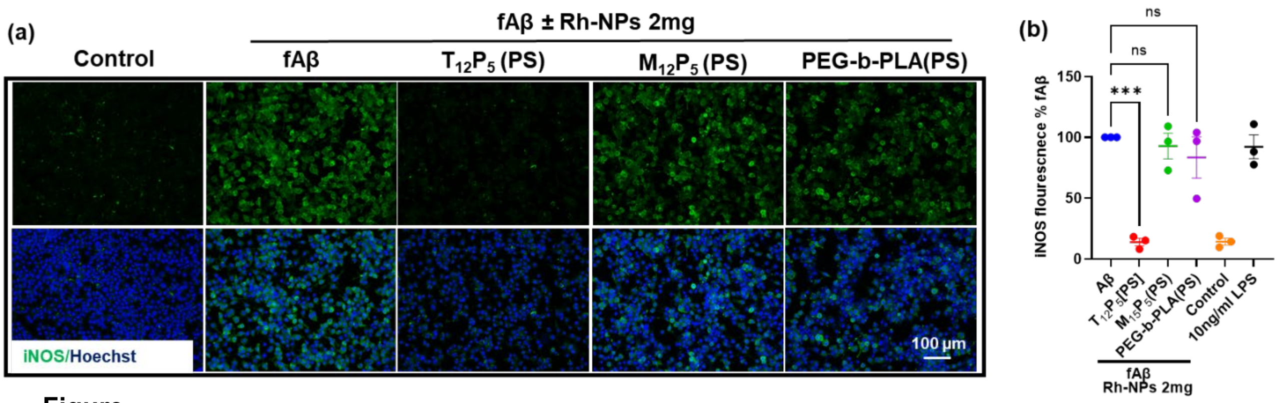


**Figure**

**S1:** Rh-NPs suppress fAβ-mediated inflammatory response in BV2 cells. BV2 microglial cells were cotreated with 20 µM fAβ in the presence or absence of Rh-NPs composed of 2mg of Rh-AMs for 24 h. (a). Fluorescence images of iNOS (green) immunoreactivity in BV2 cells. (b). Quantitative analysis of iNOS expression in BV2 cells treated with fAβ in the presence or absence of Rh-NPs**.** Data are mean ± SEM. Data analysis was conducted using one-way ANOVA followed by Dunnett's multiple comparisons test.

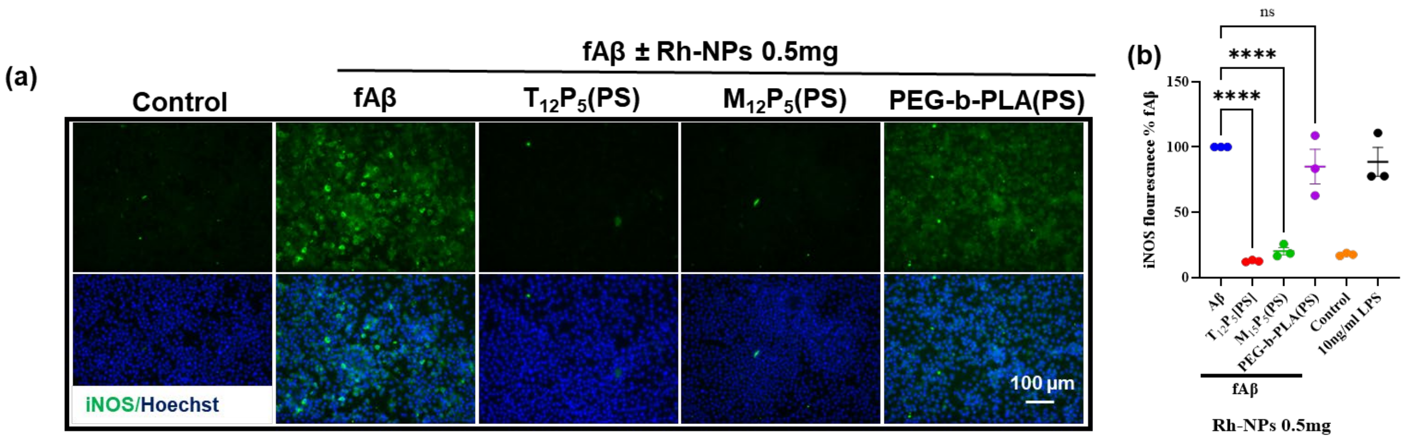


**Figure S2:** Rh-NPs suppress fAβ-mediated inflammatory response in BV2 cells. BV2 microglial cells were cotreated with 20 µM fAβ in the presence or absence of Rh-NPs composed of 0.5mg of Rh-AMs for 24 h. (a). Fluorescence images of iNOS (green) immunoreactivity in BV2 cells. (b). Quantitative analysis of iNOS expression in BV2 cells treated with fAβ in the presence or absence of Rh-NPs**.** Data are mean ±

SEM. Data analysis was conducted using one-way ANOVA followed by Dunnett's multiple comparisons test.

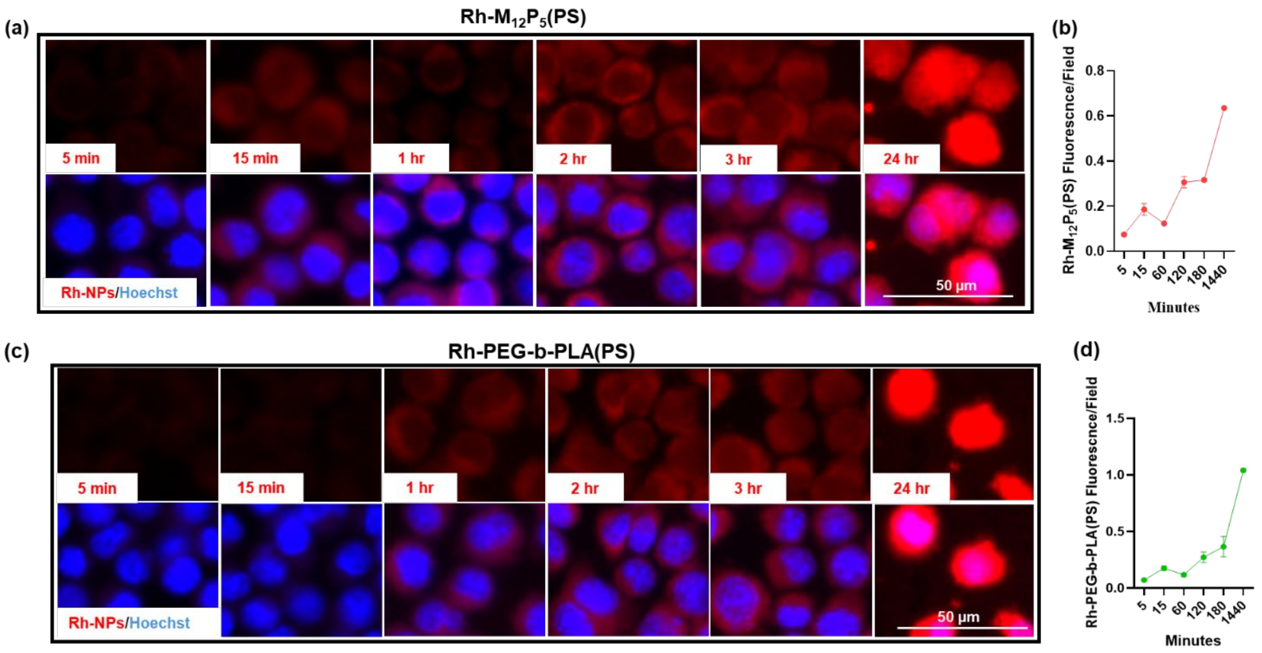


**Figure S3:** Kinetics of Cellular Uptake. (a). Fluorescence images of Rh-NPs cellular uptake. BV2 cells were incubated with Rh-$M_{12}P_5$(PS) or Rh-PEG-b-PLA(PS), then fixed with 4% PFA at different time points (5, 15, 60, 120, 180, 1440 minutes). Intracellular Rh-NPs were visualized in counterstained cells using fluorescence microscopy. (b). Fluorescence of internalized Rh-NPs was quantified at different time points and showed linear accumulation inside BV2 for up to 24 hours.

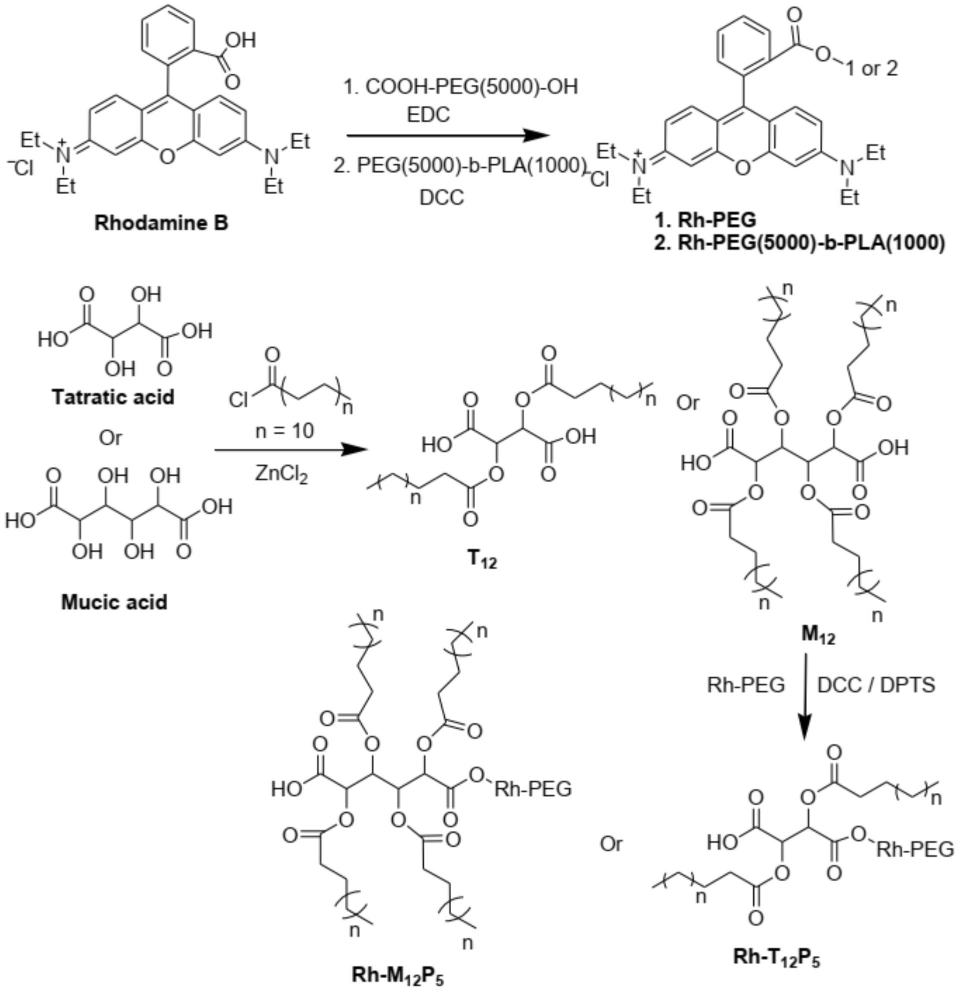


**Scheme S1.** Synthesis of Rh-AM.

# Supplemental Spectra

NMR:

$^1$H NMR ($CDCl_3$, 600 MHz) of **Rh-PEG**

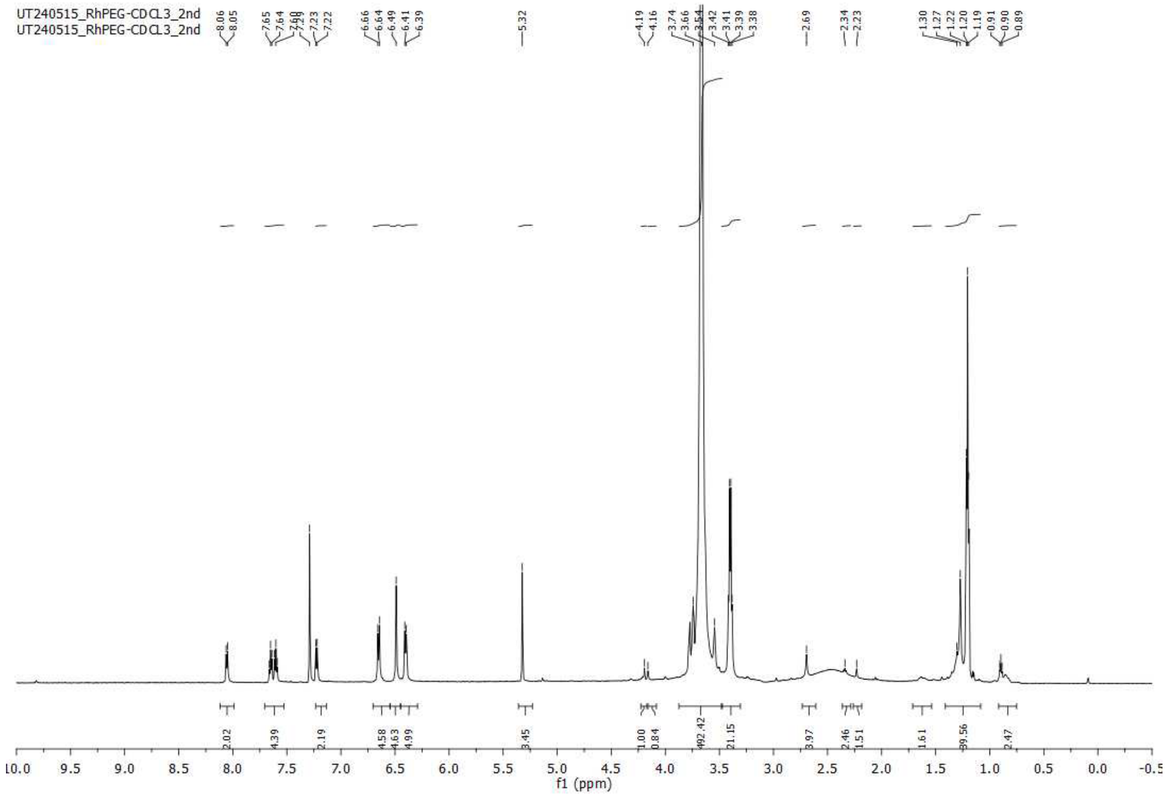


$^1$H NMR ($CDCl_3$, 600 MHz) of **Rh-T$_{12}$P$_5$**

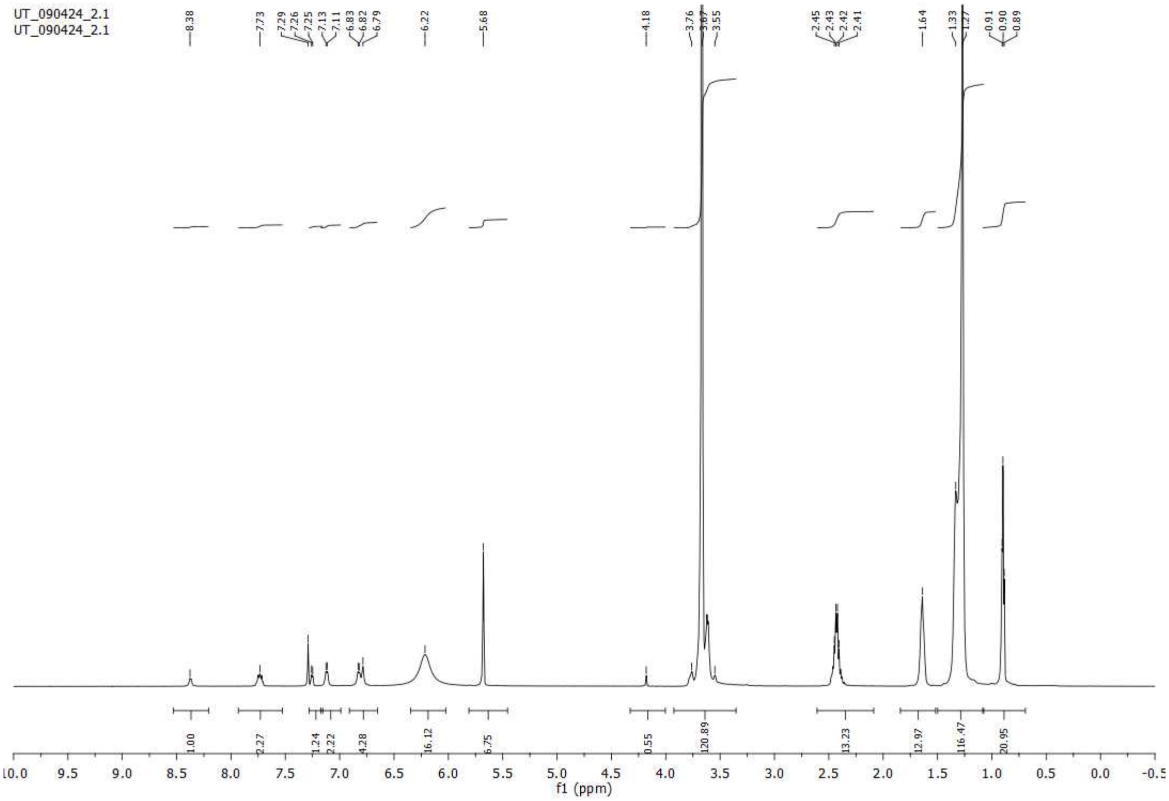


$^{13}C$ NMR ($CDCl_3$, 600 MHz) of **Rh-$T_{12}P_5$**

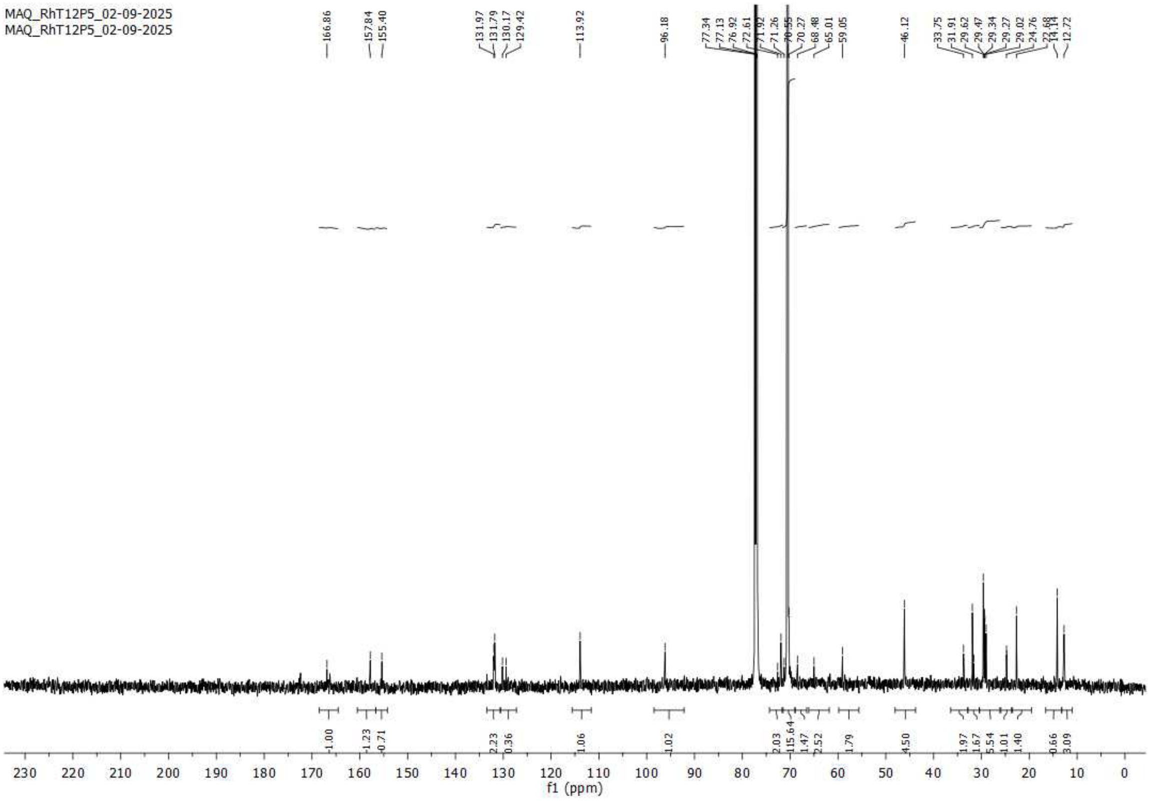


$^{1}$H NMR ($CDCl_3$, 600 MHz) of **Rh-$M_{12}P_5$**

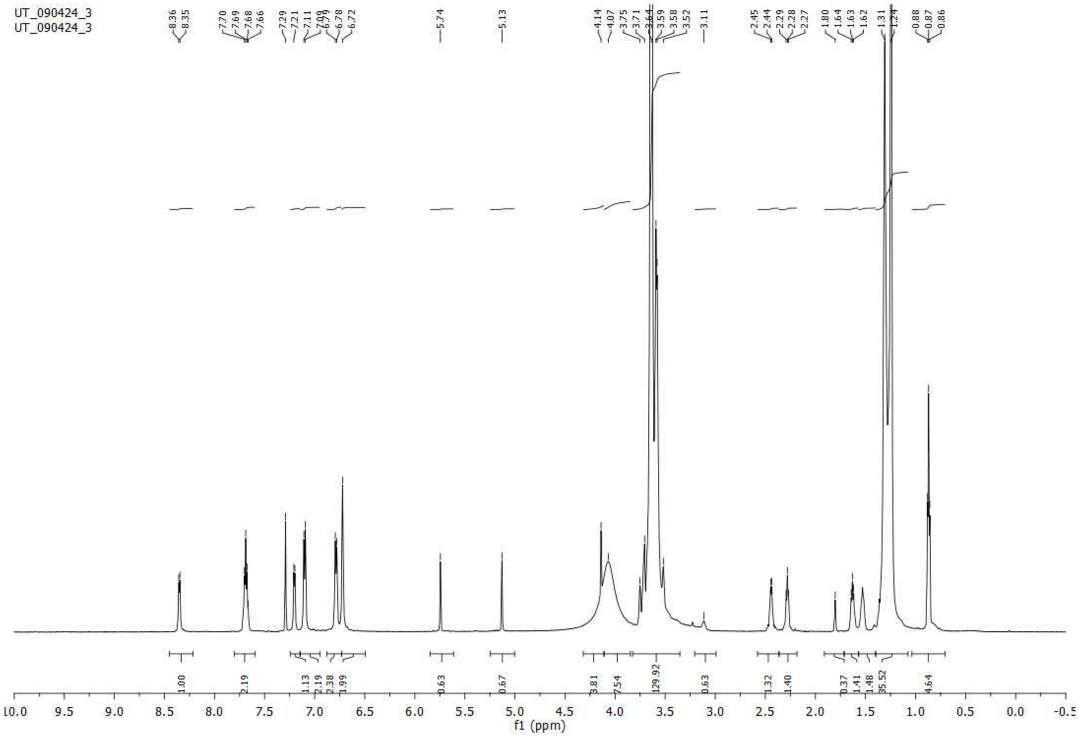


$^{13}C$ NMR ($CDCl_3$, 600 MHz) of **Rh-$M_{12}P_5$**

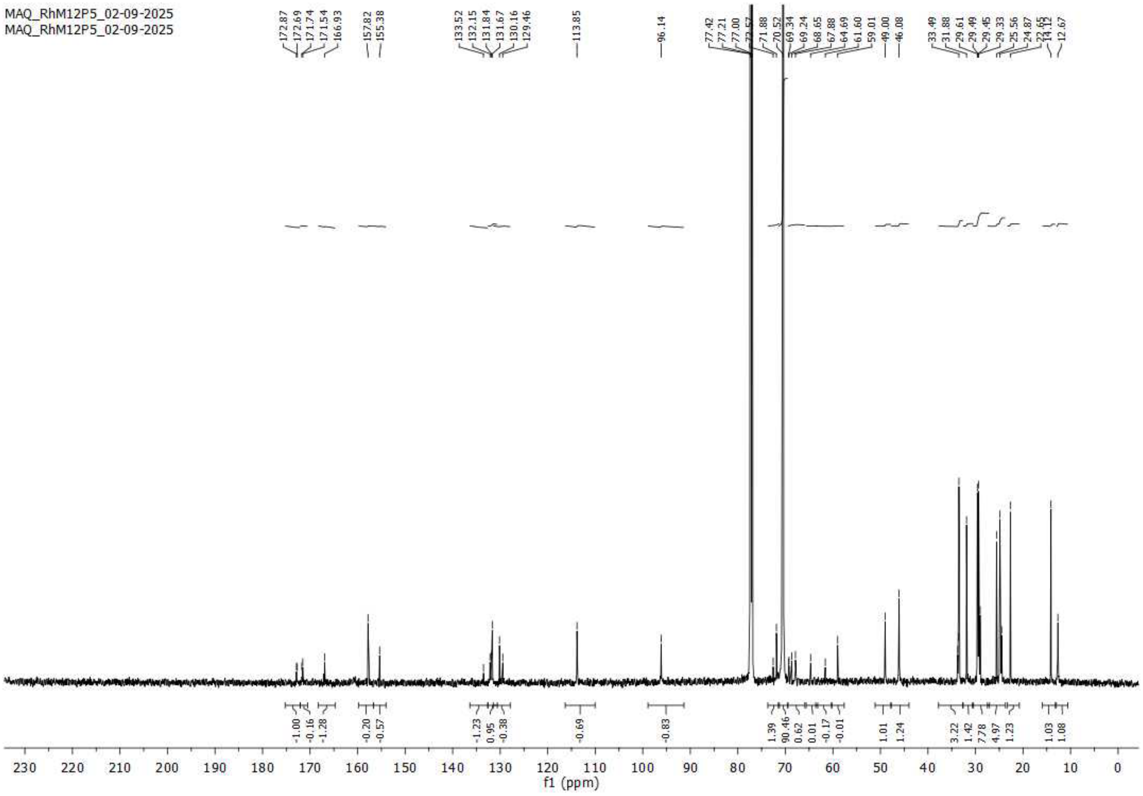

$^{1}H$ NMR ($CDCl_3$, 600 MHz) of **Rh-PEG-b-PLA**

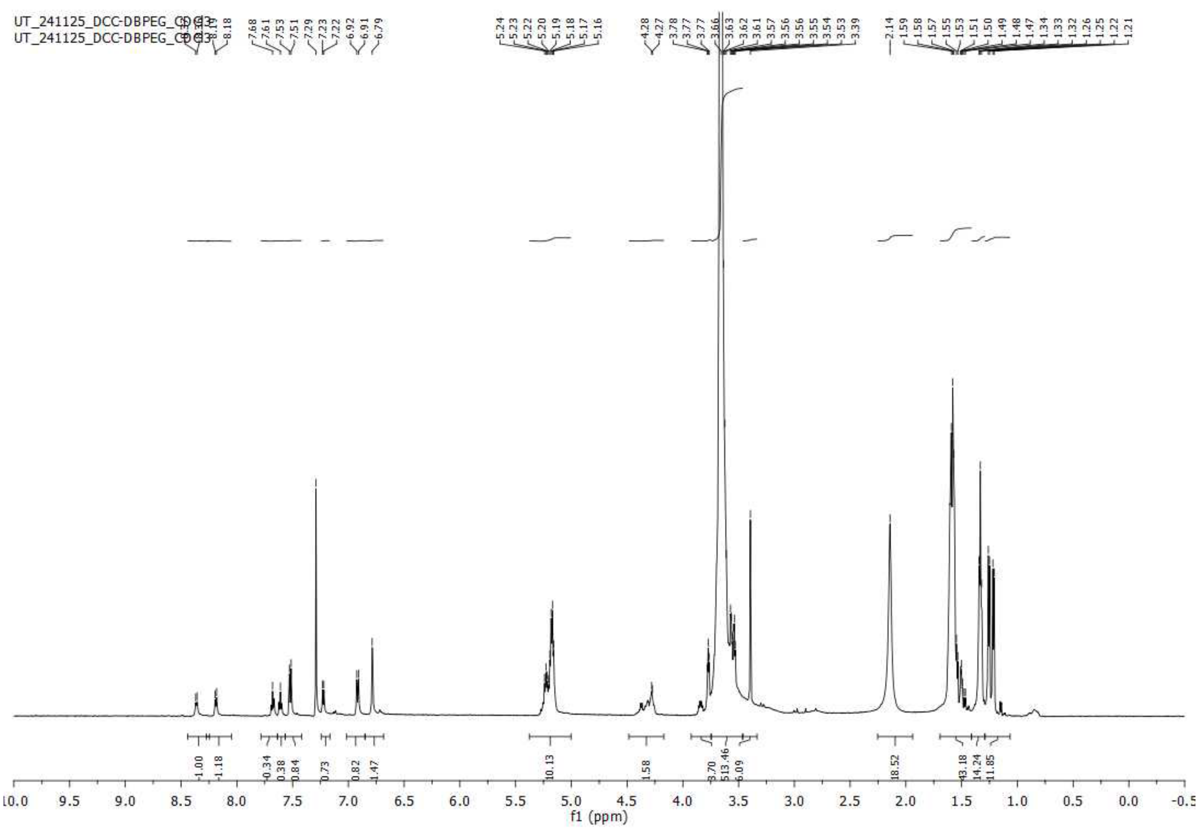

$^{13}C$ NMR ($CDCl_3$, 600 MHz) of **Rh-PEG-b-PLA**

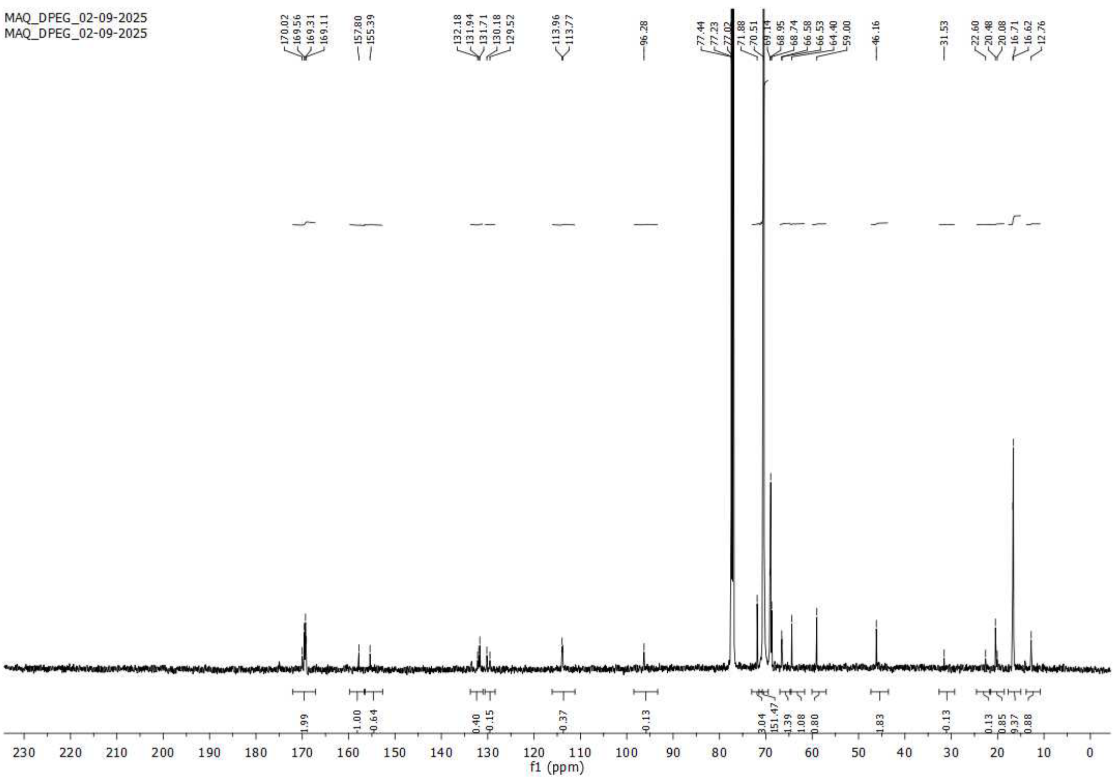


FTIR spectra :

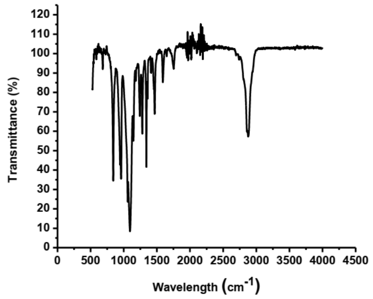


IR spectra of **Rh-T$_{12}$P$_5$**

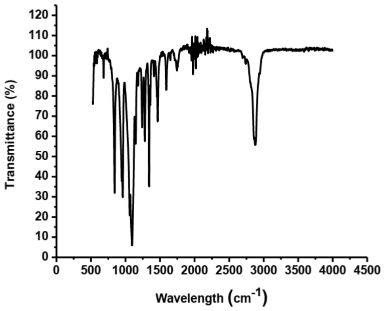


IR spectra of **Rh-M$_{12}$P$_5$**

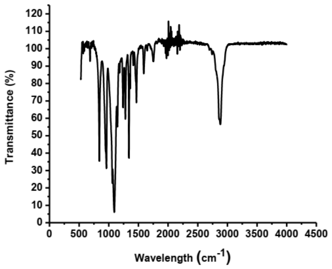


IR specta of **Rh-PEG-*b*-PLA**

UV-vis spectra:

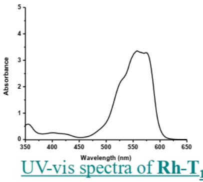


UV-vis spectra of **Rh-$T_{12}P_5$**

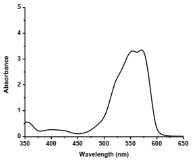


UV-vis spectra of **Rh-$M_{12}P_5$**

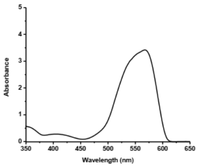


UV-vis spectra of **Rh-PEG-*b*-PLA**

Thermogravimetric analysis (TGA):

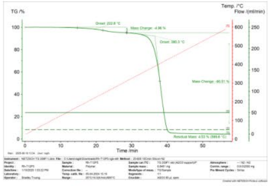


TGA of **Rh-$T_{12}P_5$**

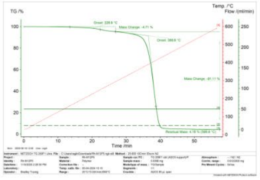


TGA of **Rh-$M_{12}P_5$**

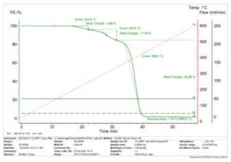


TGA of **Rh-PEG-*b*-PLA**

Differential Scanning Calorimetry (DSC):

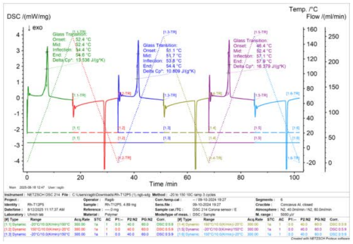


DSC of Rh-T12P5

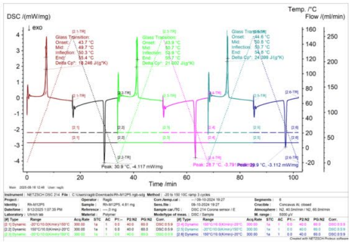


DSC of **Rh-$M_{12}P_5$**

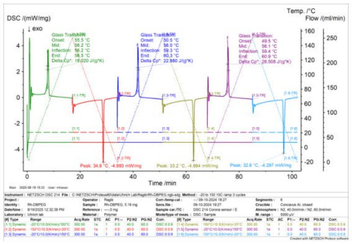


DSC of **Rh-PEG-*b*-PLA2k,**
